# Advancing musculoskeletal diagnosis and therapy: a comprehensive review of trigger point theory and muscle pain patterns

**DOI:** 10.3389/fmed.2024.1433070

**Published:** 2024-07-10

**Authors:** Tianjun Zhai, Fengyan Jiang, Yeping Chen, Jie Wang, Wei Feng

**Affiliations:** ^1^Chinese Medicine Department, The Second Rehabilitation Hospital of Shanghai, Shanghai, China; ^2^School of Rehabilitation Science, Shanghai University of Traditional Chinese Medicine, Shanghai, China; ^3^Chinese Medicine Department, Hangzhou West Lake District Beishan Street Community Health Service Center, Zhejiang, China

**Keywords:** trigger point therapy, muscle pain patterns, myofascial pain syndrome, dry needling, musculoskeletal pain management

## Abstract

Musculoskeletal disorders, especially chronic muscle pain, have a significant impact on public health, affecting millions worldwide. This review examines recent advancements in the diagnosis and management of myofascial pain, with a focus on the refined application of trigger point theory. This theory now incorporates an intricate model that blends biomechanical and neurophysiological mechanisms, essential for understanding the initiation and persistence of pain, and necessitating targeted therapeutic interventions. Utilizing a methodical approach, this paper categorizes muscle pain into three types: Muscle Belly Pain, Origin-Insertion Pain, and Referred Pain, as delineated in the most recent edition of “Myofascial Pain and Dysfunction—The Trigger Point Manual.” Such classification enhances diagnostic precision and therapeutic effectiveness by establishing a specific treatment protocol for each type of pain. The paper discusses the implications of various treatments, such as dry needling and manual therapy, which are informed by empirically derived trigger point charts. These charts are instrumental in pinpointing the exact locations of pain sources and customizing treatment plans. Moreover, this review critically assesses the evolving nature of trigger point charts and champions a holistic approach to pain management. It underscores the necessity of integrating biomechanics, kinesiology, and compensatory mechanisms to provide a comprehensive understanding that allows practitioners to address not only symptomatic pain but also the root causes of musculoskeletal disorders, thereby enhancing long-term patient care outcomes in clinical environments.

## 1 Introduction

Musculoskeletal disorders encompass a wide array of conditions that significantly impact the global population, with chronic muscle pain being a predominant issue that affects millions worldwide. These conditions not only lead to substantial personal discomfort and disability but also contribute to enormous socio-economic burdens due to healthcare costs and lost productivity (1). Among the various underlying mechanisms of musculoskeletal pain, trigger points—hyperirritable spots in skeletal muscle that are associated with palpable nodules in taut bands—play a critical role in the development and perpetuation of pain syndromes. Trigger points are often a primary factor in the onset of chronic pain, necessitating targeted treatment strategies to mitigate their effects (2).

The management of trigger points involves several therapeutic approaches, of which dry needling and manual therapy are particularly noteworthy (3). These treatments are guided by detailed trigger point charts that map the locations and characteristic referred pain patterns associated with specific trigger points. Such charts are invaluable tools in clinical settings, enabling healthcare professionals to accurately diagnose and effectively treat the complex presentations of myofascial pain. By integrating these treatment modalities within a comprehensive care plan, practitioners can significantly improve patient outcomes by directly addressing the source of pain, thus enhancing the overall quality of life for those affected by chronic musculoskeletal conditions. “*Myofascial Pain and Dysfunction: The Trigger Point Manual*,” authored by Janet Travell and David Simons, is a seminal text on myofascial pain syndrome. The book provides detailed descriptions of the locations, diagnostic techniques, and treatment strategies for myofascial trigger points, along with extensive charts that help physicians and therapists identify and address associated pains. These charts enable therapists to pinpoint trigger points linked to specific pain areas and apply manual manipulation or needling techniques to alleviate pain and improve function. However, the vast number of charts presents a memorization challenge for therapists. For example, the second edition of the manual described 255 trigger points, each associated with distinct referred pain patterns even within the same muscle, while the 2019 third edition transitioned to describing pain patterns related to muscle groups, detailing 89 muscle pain patterns with some modifications in the locations of referred pain. Recent studies have indicated that trigger points are manifestations of muscle injuries and clinical symptoms (4). Through an analysis of the muscle pain regions described in the third edition of “Myofascial Pain and Dysfunction: The Trigger Point Manual,” muscle pain patterns can be classified into Muscle Belly Pain, Origin-Insertion Pain, and Referred Pain. This classification serves to provide therapists with a rapid and precise framework for diagnosing and identifying injured muscles in the process of musculoskeletal pain rehabilitation.

The concept of trigger points was first introduced by American physician Janet Travell in 1942, who posited that these points were confined solely to the muscle belly. Her student, David Simons, expanded upon this theory by introducing the concept of “attachment trigger points” within tendons and by enhancing the precision of the trigger point maps, thereby establishing them as definitive resources in the study of trigger points (5). Trigger points typically arise from taut bands within skeletal muscles, stemming from long-standing muscle imbalances that lead to various myofascial pain syndromes. When these bands are compressed or treated with techniques such as dry needling, patients typically report characteristic radiating pain. The formation of trigger points is believed to require three criteria: a distinctly painful taut nodule, referred pain, and both local and distal muscle twitch responses upon needling and palpation (6). Simons and his colleagues hypothesized that these palpable taut bands result primarily from the excessive release of acetylcholine at neuromuscular junctions in the motor endplates, causing prolonged muscle fiber contraction, increased metabolism, and localized ischemia, which in turn trigger pain and autonomic responses, including enhanced sweating and vascular activity (7). The emergence of trigger points after muscle injury is an indicator of underlying muscle damage (8). Sensitization refers to the phenomenon where, under repeated stimulation, the nervous system’s threshold for pain perception decreases and its response to pain increases (9). This phenomenon is due to local and systemic inflammatory responses triggered by trigger points, which activate and modulate pain transmission pathways in the nervous system. In the case of myofascial trigger points, localized muscle tension and microcirculatory disturbances can trigger the release of cytokines and inflammatory mediators, sensitizing peripheral nerve endings and amplifying pain signals (10). Over time, if this local sensitization is not effectively controlled and treated, it may spread to the central nervous system, leading to what is known as central sensitization. Central sensitization involves an increased activity of neurons in the dorsal horn of the spinal cord, which can respond actively even without external stimuli, further reducing the pain threshold and leading to hyper-responses to normal tactile stimuli (such as allodynia) (11). Therefore, addressing myofascial trigger points early on, preventing further sensitization of these points, and reducing the transmission of pain signals are crucial for the early management of chronic pain. Moreover, for therapists, remembering the relevant trigger points’ referred pain within the muscles is essential.

Over the past few decades, the understanding of trigger point theory has evolved from a simplistic local muscle pain theory into a comprehensive model encompassing extensive biomechanical and neurophysiological mechanisms (12). The pioneering work of Janet Travell and David Simons has provided profound insights into trigger points, which are not only sources of muscle pain but also key factors in various chronic pain syndromes (13). With the emergence of further research on the mechanisms of action of trigger points, a deeper understanding of therapeutic approaches for these pain points has been developed, significantly impacting pain management practices.

This evolution of the theory has led to a more refined classification of myofascial pain, which is detailed in this paper by categorizing the pain patterns of 89 muscles as outlined in the third edition of “Myofascial Pain and Dysfunction—The Trigger Point Manual.” The classification divides muscle pain into Muscle Belly Pain, Origin-Insertion Pain, and Referred Pain, each based on their distinct pain features. These classifications are not only grounded in pathophysiology but also incorporate a deep understanding of etiology, allowing for more targeted treatment approaches. For instance, Muscle Belly Pain is commonly associated with overuse or injury, Origin-Insertion Pain relates to mechanical stress between the muscle and its attachment points, and Referred Pain involves more complex neural pathways issues. This structured approach ensures that treatment modalities are effectively aligned with the underlying causes of pain, enhancing the specificity and efficacy of interventions.

## 2 Muscle Belly Pain

**Figure 1:**
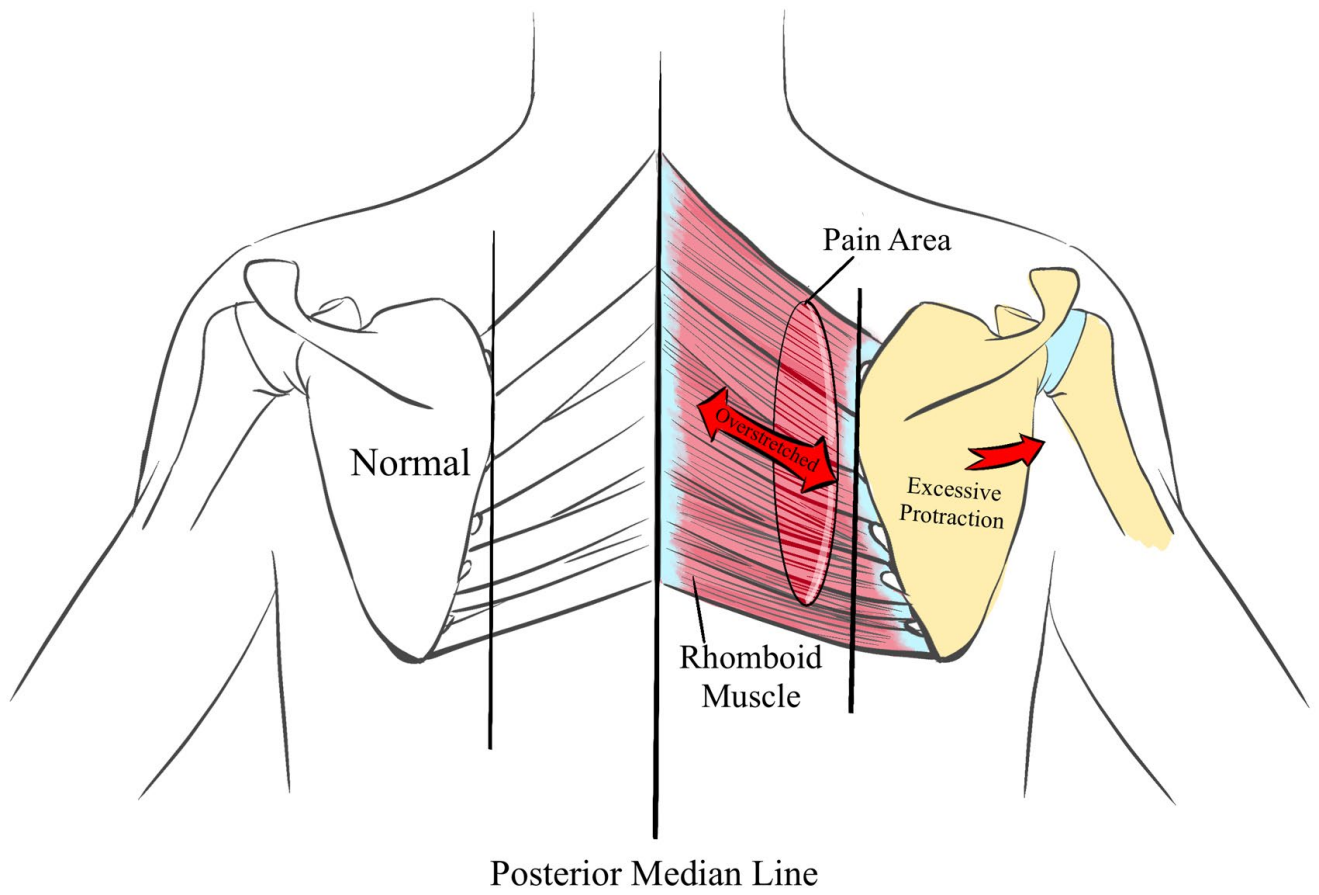
The Muscle Belly Pain in rhomboid muscles area is always associated with excessive protraction (abduction) of the scapula.

Muscle Belly Pain, which we define as pain in the belly of the muscle, a prevalent type of muscle discomfort, is often induced by external factors such as overstretching or excessive use of the muscle belly, impacting most muscles in the human body. For example, excessive protraction (abduction) of the scapula can overstretch the rhomboid muscles, leading to pain along the medial edge of the scapula (Figure 1); overuse of the biceps may cause anterior upper arm pain; and excessive stretching of the quadratus lumborum might result in lateral lumbar pain. Such pain patterns are frequently observed in clinical settings; however, therapists sometimes incorrectly identify the pain location as the primary cause, resulting in less than optimal treatment outcomes and recurrent patient conditions. In the management of muscle belly pain, it is crucial to understand the biomechanical relationships among muscles, including the interactions between agonist and antagonist muscles, the affected postures, and potential compensatory mechanisms. For instance, pain in the rhomboid muscles may be triggered by excessive stretching of the pectoralis minor and serratus anterior (14); pain in the quadratus lumborum could be caused by stretching of the contralateral quadratus lumborum (15); excessive shortening of the forearm supinator muscles may restrict forearm pronation. Such conditions during actions like reaching behind the back can lead to excessive internal rotation of the humerus, causing pain in the anterior shoulder. Therefore, therapists should focus not only on localized pain but also evaluate the overall functional state of the muscle group to ensure effective treatment outcomes. This can be achieved by relying on the relationships between agonist and antagonist muscles, muscle strength tests, and muscle length tests, to analyze whether there are functional issues with the muscles. Such as he semitendinosus and gluteus maximus muscles can both cause pain in the ischial tuberosity region when bending. By conducting length tests of the gluteus maximus and semitendinosus, one can analyze the relevant injury points. If the length of the semitendinosus decreases after knee flexion and the pain still persists, it may indicate an injury to the gluteus maximus; if the pain decreases, it suggests an injury to the semitendinosus. In conclusion, although many patients exhibit symptoms of localized muscle belly pain, addressing only the local issues often does not result in lasting therapeutic benefits.

Muscle Belly Pain is typically caused by overuse or excessive stretching of the muscles, but accurately diagnosing the specific source of this pain can be challenging, as the symptoms often resemble Muscle Belly Pain in surrounding muscles. Precise identification of the pain points and the specific location of discomfort requires detailed assessments of muscle function and strength. Understanding the biomechanical causes of muscle belly pain is crucial for selecting the appropriate treatment approach. For example, if the muscle pain is induced by specific movements or improper postures, appropriate physical therapy and adjustments to the way movements are performed can significantly improve the patient’s symptoms. Additionally, educating patients on how to avoid activities that may trigger the pain forms an integral part of the treatment strategy.

## 3 Origin-Insertion Pain

**Figure 2:**
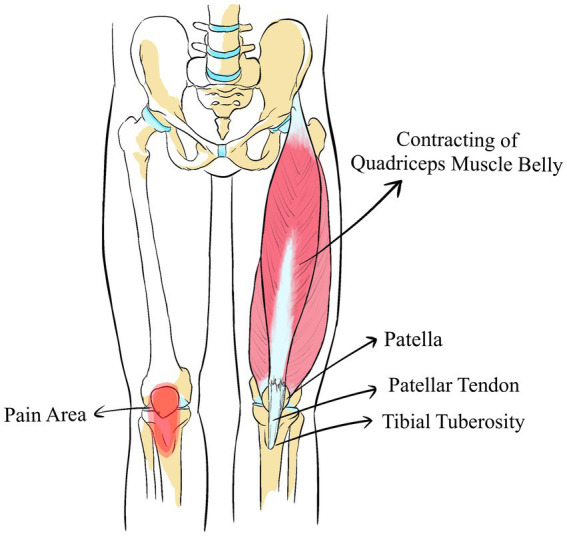
The Origin-Insertion Pain of quadriceps lead to pain extending from the patella to the tibial tuberosity.

We define Origin-Insertion Pain as the pain occurring at the attachment points of muscles following repeated overuse. Muscle contractions move bones by pulling on the tendons, and the repetitive contractions can easily lead to pain in their attachment areas. For instance, excessive use of the quadriceps can lead to pain extending from the patella to the tibial tuberosity (16) (Figure 2). Similarly, tennis elbow often involves the extensor carpi radialis longus, extensor carpi radialis brevis, and supinator muscles, all of which connect to the lateral epicondyle of the humerus (17). Beneath the medial malleolus, the flexor retinaculum encompasses the posterior tibial muscle, the long flexor muscle of the toes, and the long flexor muscle of the metatarsus, which likely contribute to pain in this region (18). Such pain is frequently linked to muscle overuse, where repetitive actions cause tendons to persistently pull at their bony attachment points, thereby increasing local stress. For example, chronic anterior knee pain might stem from the hyperextension and excessive strain on the quadriceps, enlarging the tibial tuberosity (19). Plantar fasciitis could result from the repeated pulling of the quadratus plantae or plantar fascia on the calcaneal tuberosity, causing heel spurs (20). de Quervain tenosynovitis may be related to friction between the sheaths of the abductor pollicis longus and the extensor pollicis brevis and the radial styloid, causing localized swelling (21). Detecting changes in these bony landmarks through palpation or imaging provides a clinical basis for evaluating muscle overuse. In clinical practice, palpating these specific areas is an effective method to determine muscle overuse, as many symptoms are manifest at these anatomical sites.

Origin-Insertion Pain involves mechanical stress between muscles and their attachment points, making this type of pain challenging to diagnose due to its specific location. Such pain points may become inflamed due to muscle tension or minor tears, necessitating precise imaging studies and physical examinations for accurate identification. Effective treatment of Origin-Insertion Pain requires alleviating tension in the muscles and tendons to restore their normal function. Treatment modalities may include localized injections, physical therapy, or specific stretching and strengthening exercises. Proper treatment not only alleviates pain but also helps prevent future injuries.

## 4 Referred Pain

Referred pain, historically discussed as either visceral or somatic, is the discomfort perceived in regions distant from the source. Visceral referred pain arises from damage to internal organs, affecting areas served by the corresponding nerve roots of the sensory nerves (22). Spinal referred pain, on the other hand, stems from stimulation of structures like ligaments, intervertebral disks, and facet joints, leading to pain elsewhere (23). In the context of trigger point theory, it is posited that each muscle may exhibit a unique pattern of referred pain, although the vast number of referred pain maps implies that a single muscle could exhibit multiple referred pain manifestations. These referred pains are classified based on their origin into Peripheral Nerve Referred Pain, Same-root Nerve Radicular Pain, and Special Referred Pain, each reflecting different underlying mechanisms.

### 4.1 Peripheral Nerve Referred Pain

**Figure 3:**
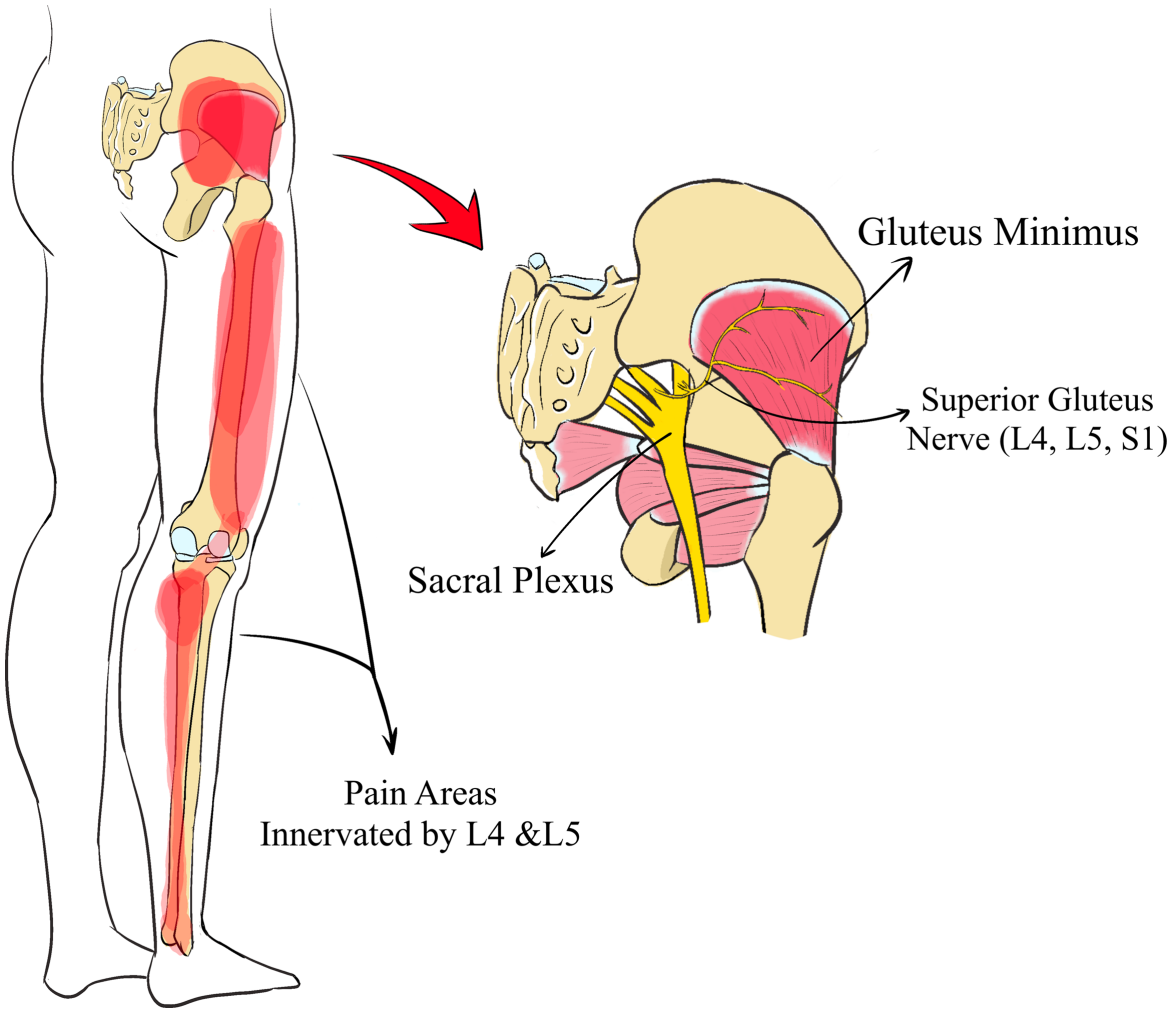
The Peripheral Nerve Referred Pain of scalene muscle.

Peripheral nerve involvement in musculoskeletal pain is associated with nerves traversing within muscles. When these nerves are activated through needling or manual techniques, they cause pain to radiate to the areas they innervate. For example, damage to the iliopsoas muscle may lead to pain in the anterior thigh and groin, which cannot be fully explained by merely examining the muscle’s origin and insertion points (24). However, considering the impact on nerves such as the iliohypogastric, ilioinguinal, and femoral nerves traversing the iliacus clarifies the pain pattern. The iliohypogastric nerve’s abdominal branch and the ilioinguinal nerve control the groin area, while the femoral nerve covers the anterior thigh, making the resulting pain patterns more comprehensible. Similarly, damage to the anterior scalene muscle often causes referred pain to the chest’s front, the inner border of the scapula, and even the thumbs and little fingers (25). This pain pattern is linked to the nerves passing behind or between the scalenes, including branches of the brachial plexus, the long thoracic nerve, the dorsal scapular nerve, the radial nerve, and the ulnar nerve (Figure 3). Consistency in the nerves running through various muscles may lead to shared pain areas, as seen with the long head of the triceps, teres minor, and teres major, which, together with the humerus, form the quadrilateral space for the axillary nerve that affects the deltoid area it innervates (26). In clinical treatment, effectively addressing related pain first involves identifying the nerves that innervate the affected area. Treatment can then proceed by needling or manually releasing the muscles through which these nerves pass, directly alleviating the pain in the targeted region.

### 4.2 Same Nerve Root Radicular Pain

**Figure 4:**
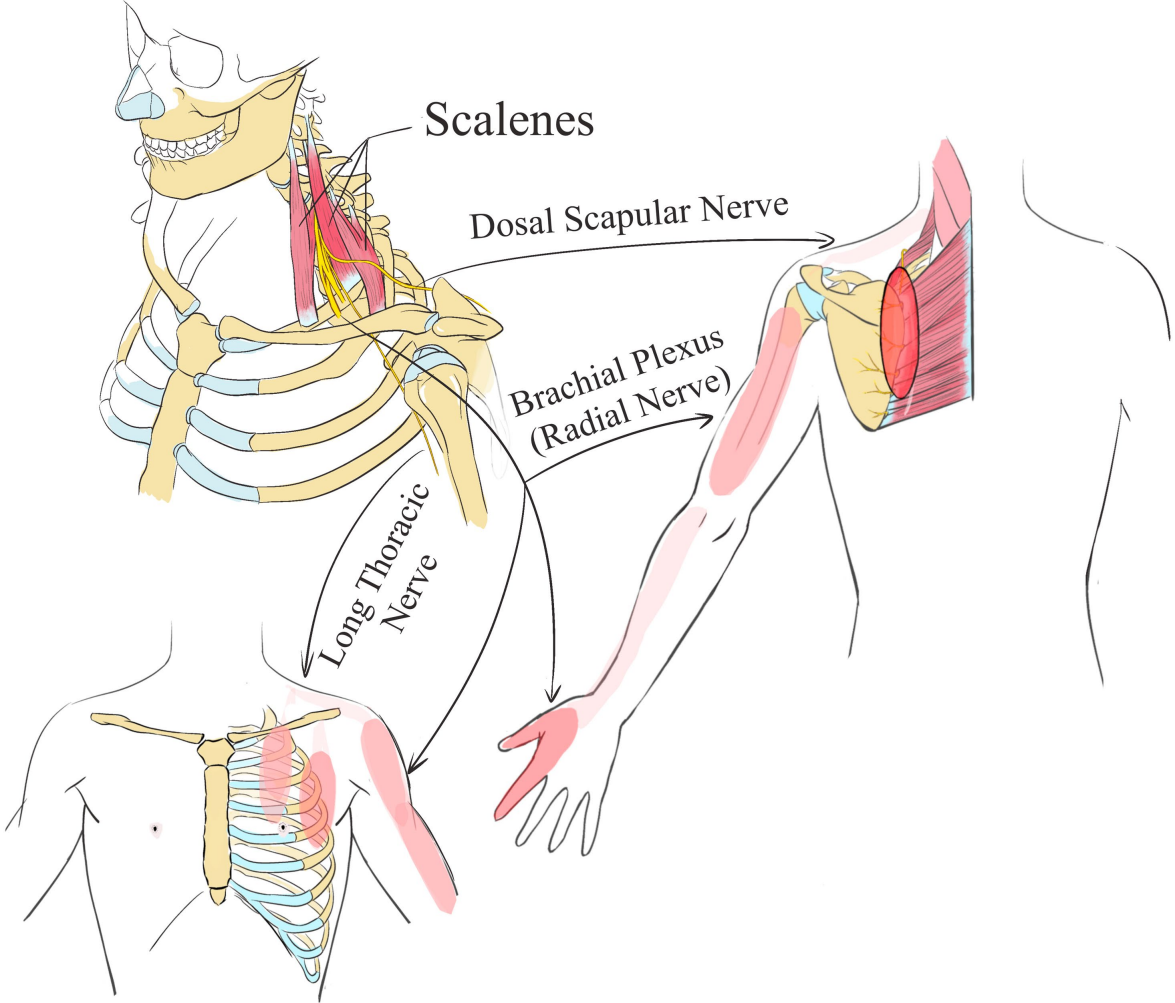
The Same Nerve Root Radicular Pain of gluteus minimus.

The second type of referred muscle pain involves shared same nerve root radiation, a phenomenon where prolonged stimulation of a branch nerve can affect another branch via the same nerve root, resulting in corresponding nerve stimulation. Muscle contractions, regulated by nerve control, often coincide with functional abnormalities in the peripheral nerves. According to the theory of axonal flow, these abnormalities influence the nerve roots and subsequently radiate through the shared nerve root, affecting the corresponding dermatome area controlled by these nerves. For example, damage to the infraspinatus, innervated by the upper scapular nerve (C_5_, C_6_), may induce pain in the long head of the biceps brachii region at the anterior shoulder, governed by the musculocutaneous nerve (C_5_, C_6_, C_7_). If the infraspinatus is compromised, the upper scapular nerve is impacted, altering its internal blood supply and increasing tension in the C_5_ and C_6_ nerve roots, which then affects the musculocutaneous nerve, leading to biceps pain. Similarly, an injury to the gluteus minimus, which influences the lateral areas of the thigh and lower leg, can cause pain in these regions (27). The gluteus minimus is controlled by the superior gluteal nerve (L_4_, L_5_, S_1_), and the lateral parts of the thigh and lower leg are also innervated by the L_4_ and L_5_ nerves (Figure 4). When needling is performed in the gluteus medius and gluteus minimus, patients may report radiating pain in the lateral thigh and lower leg. This type of Same Nerve Root Radicular Pain often manifests not as pain in the muscle area through which the nerve passes but rather in the area controlled by the nerve roots, which makes it susceptible to misdiagnosis as a nerve root disorder. As previously mentioned, damage to the gluteus minimus often presents as pain in the lateral aspect of the lower leg and can be incorrectly diagnosed as conditions such as spinal stenosis or lumbar nerve root compression.

Peripheral Nerve Referred Pain involvement primarily targets the nerve trunk, which may be concentrated in a specific area or throughout the entire region innervated by the peripheral nerve. This occurs as the nerve exits the muscle, and stimulation of the muscle can lead to referred pain in the area innervated by that nerve. On the other hand, Same Nerve Root Radicular Pain associated with the same nerve root involves the nerve root itself. This type of pain emerges when there is dysfunction in the motor fibers of the nerve root, manifesting as muscular issues. Subsequently, stimulation of the affected muscles can lead to pain in the areas served by the sensory fibers of that nerve root. This pain typically encompasses a broader area and may affect multiple body parts innervated by the same nerve root.

### 4.3 Special Referred Pain

We define the third type of muscle referred pain as “Special Referred Pain,” Which is characterized by an absence of a clear pattern or established mechanism of referral. This category includes instances such as an injury to the soleus muscle causing referred pain to the same side of the cheek area, an injury to the subscapularis muscle resulting in pain on the dorsal side of the wrist, and diaphragm injury leading to pain above the first rib. These examples do not conform to the conventional theories of peripheral nerve involvement or shared nerve reflexes and lack consistent patterns. As a result, they are classified under special referred pain, distinguished by their unique and not yet fully understood nature.

The diagnosis of Referred Pain is particularly complex because the pain sensation often appears at locations far from the actual trigger points. This requires physicians to have a comprehensive understanding of the pain transmission pathways related to the involved nerves and muscle groups to accurately identify the source of pain. The treatment of Referred Pain focuses on addressing the underlying neural and muscular issues and may include techniques such as dry needling, massage, and other manual therapies directly targeting the trigger points. This treatment not only requires precise diagnosis but also necessitates personalized treatment plans tailored to the specific pain patterns and the overall condition of the patient.

Statistical analysis of The third edition of “Myofascial Pain and Dysfunction: The Trigger Point Manual” presents muscle distribution and associated pain patterns. It reports that the majority of muscles manifest three primary types of pain: Muscle Belly Pain (85.4%), Origin-Insertion Pain (80.9%), and Referred Pain (59.5%) (Table 1). This detailed distribution underscores the pervasive nature of these pain patterns across different muscle groups. Regarding Referred Pain, Peripheral Nerve Referred Pain predominantly affects proximal limbs and the spine. In contrast, Same-Root Nerve Reflex pain typically occurs around the shoulders, back, pelvis, and hips, with instances in the forearm, wrist, and hands being rare. This observation supports the theoretical foundation for addressing distal joint discomfort via interventions at the spine or proximal joints. For instance, wrist pain might stem from complications in the forearm, elbow, shoulder, or cervical spine, necessitating a detailed assessment to pinpoint the exact location of the injury based on the presented pain.

**Table 1:** Table of muscle pain patterns by body part.

	Number of muscles	Muscle Belly Pain	Origin-Insertion Pain	Referred Pain	Same Nerve Root Radicular Pain
				Peripheral Nerve Referred Pain	
Head, Face, and Neck	11	8	9	8	2
Upper Back and Shoulder	13	9	11	4	8
Forearm, Wrist, and Hand	18	14	8	6	0
Trunk and Pelvis	15	13	14	5	8
Hip, Thigh, and Knee	16	16	15	4	3
Calf, Ankle, and Foot	16	16	15	4	1
Total	89	76 (85.4%)	72 (80.9%)	31 (34.8%)	22 (24.7%)

## 5 Discussion

In recent years, trigger point theory has increasingly been recognized, though the corresponding referred pain maps remain underdeveloped. While this theory adopts a holistic approach to developing treatment plans, it neglects the influences of kinesiology, biomechanics, and compensatory mechanisms. Consequently, this article methodically categorizes muscle pain patterns into Muscle Belly Pain, Origin-Insertion Pain, and Referred Pain. Such classification is essential for the rapid diagnosis and effective treatment of pain arising from muscle injuries in clinical environments, facilitating more precise and targeted therapeutic interventions. Our theoretical framework expands the traditional concept of myofascial trigger points to encompass a comprehensive understanding of the interactions between muscle groups and the nervous system, emphasizing holistic rather than isolated point interactions. This approach significantly improves diagnostic accuracy by accounting for nerve pathways within muscle structures, thereby enabling more precise localization of pain sources—a crucial advantage in managing complex myofascial pain where conventional methods may be inadequate. Additionally, our theory extends therapeutic strategies beyond traditional treatments such as dry needling and massage, incorporating diverse physical and rehabilitative therapies, including functional electrical stimulation and targeted exercise regimes aimed at comprehensive neuromuscular adjustment. This not only enhances treatment outcomes but also promotes effective preventative measures through a deeper understanding of muscle and nerve interactions. Moreover, it opens new research avenues into the underlying causes of myofascial pain, especially regarding the role of the nervous system, thereby facilitating the development of novel diagnostic and treatment technologies and improving patient rehabilitation outcomes.

### 5.1 Neuromuscular mechanisms in trigger point therapy

Trigger points, located within muscle fibers, create highly sensitive and painful areas, often due to localized ischemia and energy crises. This condition results in sustained muscle contraction knots. The continuous muscle contractions hinder blood flow, thereby reducing oxygen and nutrient delivery and impeding waste removal (28). This process leads to an accumulation of metabolic waste products such as lactic acid, enhancing pain sensitivity under hypoxic conditions. Ischemia and hypoxia activate chemoreceptors and nociceptors, which then produce and release inflammatory mediators, including prostaglandins and cytokines. These mediators sensitize or activate adjacent pain nerve endings, thus triggering pain signals. Additionally, the chemically reactive environment at the trigger point site promotes the release of neurotransmitters like serotonin and norepinephrine. These neurotransmitters not only modulate pain transmission but also contribute to the cycle of sustained muscle contraction and pain (29).

Furthermore, trigger points can exacerbate pain sensitivity through central sensitization processes. In these processes, spinal level pain neurons undergo both functional and structural changes, becoming more responsive to pain stimuli originating from trigger points. This sensitization is not confined solely to the local area but may also extend to the brain, intensifying the overall perception and experience of pain (30).

Dry Needling directly stimulates trigger points, activating local muscle fibers which then induce depolarization and muscle twitch responses. These responses help disrupt the sustained contraction within the muscle, enhance local blood flow, and decrease ischemic pain (31). Dry needling also affects pain transmission pathways in the spinal cord and brain, activating the endogenous analgesic system and releasing endorphins and other natural pain relievers (32). Massage alleviates muscle tension by applying manual pressure and kneading to trigger points, which improves blood circulation and promotes the elimination of metabolic waste (13). The mechanical stimulation delivered during massage transmits signals through mechanoreceptors, inhibiting pain signal transmission and providing analgesic effects. Additionally, tactile stimulation during massage activates the body’s endogenous analgesic mechanisms, further enhancing pain relief (33). Electrical Stimulation stimulates muscles and nerves with electrical currents, triggering muscle contractions and nerve activation (34). It disrupts pain signal transmission and inhibits pain perception through the gate control theory. Electrical stimulation also elevates pain thresholds, activates the endogenous analgesic system, and promotes the release of pain-relieving substances (35).

These therapeutic techniques underscore the extensive neuromuscular interactions involved in treating trigger points, emphasizing the necessity of understanding muscle referred pain from a neurological perspective.

### 5.2 Completeness of trigger point charts

Trigger point charts represent a form of empirical medicine, mainly developed by injecting a 2% saline solution and anesthetics into trigger points and subsequently refining the referred pain pathways based on patient descriptions (28). However, this approach does not eliminate the subjectivity of participants or the potential influence of operators when patients are unable to specify their pain locations precisely. The trigger point charts are continuously evolving; for instance, whereas the pain pattern for the diaphragm in the second edition was localized only to the diaphragm and rib area, the third edition extends this to include pain above the first rib. Additionally, the second edition lacked a pain map for the sternocleidomastoid affecting the lateral and anterior neck areas, which was included in the third edition. Clinically, discrepancies with the trigger point charts are common, such as when needling the iliopsoas at the lesser trochanter might lead patients to experience referred pain to the groin, anterior thigh, and inner thigh—areas not described in the trigger point charts. Similarly, needling the piriformis muscle may cause an electric shock sensation extending from the posterior thigh to the sole of the foot, which are areas not depicted in the maps. Moreover, needling the internal and external obturator muscles often results in patients reporting radiating pain in the inner thigh, controlled by the obturator nerve, though the maps indicate radiating pain above the hamstring on the posterior thigh. These examples demonstrate that the trigger point maps are not yet flawless and do not comprehensively represent muscle pain patterns, necessitating ongoing refinement of referred pain maps. Nevertheless, based on the systematic and predictable muscle pain patterns of Muscle Belly Pain, Origin-Insertion Pain, and Referred Pain outlined in this article, it is feasible to develop muscle pain pattern charts that provide a theoretical basis for clinicians to diagnose injured muscles based on the location of a patient’s pain, thereby enhancing clinical efficiency.

### 5.3 Limitations of trigger point theory

Trigger point theory suggests that muscle injury is the primary source of pain, primarily concentrating on local muscle dynamics, yet often disregards the impacts of biomechanics, kinesiology, and compensatory mechanisms on the human body. While manual and dry needling therapies effectively alleviate most pain, recurrence is frequent, as these treatments fail to address the root causes of muscle injuries. For example, treating pain along the medial border of the scapula typically involves needling the rhomboid muscles or the neck area. The third edition of the relevant manual advises treatment of the scalene and levator scapulae muscles due to the passage of the dorsal scapular nerve, a branch of the brachial plexus, through these muscles. However, such an approach often overlooks essential biomechanics and kinesiology. Pain in the rhomboid muscle area usually results from scapular abduction, which over-stretches the medial muscles, primarily caused by the tension and pulling of the serratus anterior and pectoralis minor muscles. Consequently, treatment should primarily target these muscles instead of merely addressing the pain in the dorsal area. Similarly, pain at the front of the knee from a quadriceps injury should prompt consideration of the iliopsoas’ weakness and compensatory hip flexion by the rectus femoris, or an anterior pelvic tilt leading to knee hyperextension. Thus, clinical application of trigger point theory in diagnosing muscle injuries should incorporate an understanding of biomechanics, kinesiology, and compensatory processes, as the underlying causes of local muscle damage are often intricate and necessitate a comprehensive clinical approach. Additionally, chronic musculoskeletal pain is usually not caused by a single trigger point or muscle issue but involves a complex interplay of biological, psychological, and social factors. Biologically, the interactions among nerves, muscles, bones, and soft tissues, as well as potential degenerative changes, form the basis of pain. Psychological factors such as stress, anxiety, and depression play significant roles in pain perception and the chronicity of the condition. Social factors, including occupational stress and lifestyle habits, also impact the progression and severity of the pain. This multifactorial nature necessitates the adoption of more comprehensive and personalized treatment strategies in future clinical practice (36).

### 5.4 The importance of muscle pain patterns in clinical diagnosis and treatment

In the assessment of musculoskeletal pain, conducting extensive specialized examinations is crucial. However, novice therapists might overlook certain influential factors or rare causes during these assessments, which can lead to missed or incorrect diagnoses. By precisely analyzing the patient’s described area of pain and correlating it with established muscle pain patterns—Muscle Belly Pain, Origin-Insertion Pain, and Referred Pain—it becomes possible to swiftly pinpoint the specific muscle responsible for the pain. This approach also aids in assessing the severity of the muscle injury based on the results of specialized examinations, thereby facilitating effective treatment measures that yield positive clinical outcomes. Modern pain treatment requires therapists to not only focus on localized symptoms but also to consider the origins of pain from a holistic perspective. The pain patterns of Muscle Belly Pain, Origin-Insertion Pain, and Referred Pain not only address local symptoms but also illustrate how pain can spread from proximal to distal parts of the body. Research indicates that approximately 85% of referred pain propagates from proximal to distal locations (37). For example, pain in the lateral epicondyle of the humerus may stem from issues in the triceps or supraspinatus muscles (38); pain on the outer side of the calf may result from damage to the piriformis muscle (39). Viewing the upper limbs and cervical spine as one integrated system, and the lumbar spine and lower limbs as another, highlights the critical interaction between muscles within these systems for understanding pain. Therefore, acknowledging the overall transmission of pain in the diagnosis and treatment of chronic pain is essential, as it facilitates a more comprehensive approach to addressing pain issues.

### 5.5 The importance of comprehensive assessment for pain

The evaluation of pain in patients should not focus solely on individual muscles or trigger points, but instead incorporate a series of integrated assessment strategies to thoroughly analyze the patient’s movement and biomechanical properties. Firstly, movement assessment forms the core of functional evaluation. By observing patients performing specific movements or daily activities, we can identify potentially harmful movement patterns that may cause or exacerbate pain. For instance, patients might be asked to perform squats, walk, or engage in other specific activities to observe muscle coordination, joint mobility, and pain trigger points during these movements (31). Next, biomechanical assessment aids in understanding how a patient’s physical structure impacts their pain. This involves analyzing body symmetry, joint alignment, and the balance between muscle strength and flexibility. Through detailed assessment of these factors, we can identify structural and functional issues that contribute to pain, such as excessive muscle tension, uneven joint pressure, or instability. Additionally, we consider other relevant factors, including the functional status of the nervous system and psychosocial factors, which can affect a patient’s perception of pain and response to treatment (40). By utilizing neurofunctional tests and psychological assessment tools, we can gain a more comprehensive understanding of the multidimensional complexity of pain and devise more effective treatment strategies. During these assessments, we employ a variety of tools and technologies, including but not limited to dynamic electronic devices, pressure sensors, and visual analysis software. These tools enable us to precisely measure and record data during the assessment process, providing quantitative feedback to help optimize treatment plans.

In summary, functional assessment is a dynamic and interactive process that requires a holistic consideration from multiple perspectives to deeply understand and address pain issues. Through this approach, we can offer patients personalized treatment plans that not only alleviate pain but also enhance their overall function and quality of life.

### 5.6 Integrated multidisciplinary strategies for chronic pain management

Chronic pain represents a multifaceted clinical challenge that involves intricate interplays between biological, psychological, and sociological factors (36). Effective management of chronic pain requires the synergistic integration of diverse professional expertise, including that of physical therapists, neurologists, psychologists, orthopedic surgeons, rehabilitation specialists, and pain management clinicians. Such collaborative efforts enable a comprehensive assessment of the root causes of pain, facilitating the formulation of tailored treatment strategies designed to optimize patient outcomes.

Critical to this approach is the meticulous assessment of trigger point pain, which necessitates accurate localization of pain points, characterization of their nature, and evaluation of their impact on the patient’s quality of life (41). This assessment process encompasses an extensive collection of medical history, thorough physical examinations, detailed functional assessments, and comprehensive neurological evaluations. The multidisciplinary treatment modalities for addressing trigger point pain might include physical therapy interventions such as dry needling, thermotherapy, cryotherapy, and electrical stimulation; pharmacological management using anti-inflammatory agents, muscle relaxants, or antidepressants; psychological interventions, notably cognitive behavioral therapy; and rehabilitation programs that feature personalized exercise regimens and lifestyle adjustments. Additionally, educational and self-management strategies are employed to empower patients (42).

This cohesive and coordinated treatment paradigm not only directly targets the sources of pain but also significantly aids patients in navigating the psychological and social complexities associated with chronic pain. By leveraging the combined expertise of a multidisciplinary team, the effectiveness of the therapeutic interventions is markedly enhanced, offering patients a comprehensive pathway toward sustained recovery. This approach is aligned with the principles of patient-centered care, which prioritize individual health needs and preferences in the design and implementation of healthcare strategies.

### 5.7 Summary

Categorizing muscle pain into Muscle Belly Pain, Origin-Insertion Pain, and Referred Pain not only enhances the efficiency of diagnosing and treating musculoskeletal pain but also lays a theoretical foundation for the further development of trigger point theory and charts. Additionally, considering a variety of factors that contribute to muscle injuries is crucial for managing chronic pain, which aids in comprehensively addressing pain issues and promotes overall advancements in musculoskeletal medicine.

## Data availability statement

The original contributions presented in the study are included in the article/supplementary material, further inquiries can be directed to the corresponding author.

## Author contributions

TZ: Writing – original draft. FJ: Methodology, Visualization, Writing – review & editing. YC: Writing – review & editing. JW: Writing – review & editing. WF: Conceptualization, Writing – review & editing.
